# Genetic pathways controlling inflorescence architecture and development in wheat and barley

**DOI:** 10.1111/jipb.12732

**Published:** 2019-02-01

**Authors:** Adam Gauley, Scott A. Boden

**Affiliations:** ^1^ Department of Crop Genetics John Innes Centre Norwich Research Park Norwich NR4 7UH United Kingdom

## Abstract

Modifications of inflorescence architecture have been crucial for the successful domestication of wheat and barley, which are central members of the *Triticeae* tribe that provide essential grains for the human diet. Investigation of the genes and alleles that underpin domestication‐related traits has provided valuable insights into the molecular regulation of inflorescence development of the *Triticeae*, and further investigation of modified forms of architecture are proving to be equally fruitful. The identified genes are involved in diverse biological processes, including transcriptional regulation, hormone biosynthesis and metabolism, post‐transcriptional and post‐translational regulation, which alter inflorescence architecture by modifying the development and fertility of lateral organs, called spikelets and florets. Recent advances in sequencing capabilities and the generation of mutant populations are accelerating the identification of genes that influence inflorescence development, which is important given that genetic variation for this trait promises to be a valuable resource for optimizing grain production. This review assesses recent advances in our understanding of the genes controlling inflorescence development in wheat and barley, with the aim of highlighting the importance of improvements in developmental biology for optimizing the agronomic performance of staple crop plants.

## Graphical abstract

This review summarizes the recent advances on the genetic regulation of inflorescence architecture in the *Triticeae* tribe, including the staple crops of wheat and barley. We focus on the key domestication related traits, and recent studies that have used modified forms of inflorescence architecture to identify genes regulating spikelet and floret development.

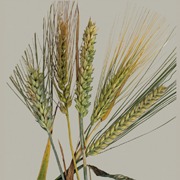

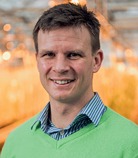

Scott A. Boden

**Edited by:** Thorsten Schnurbusch, Leibniz Institute of Plant Genetics and Crop Plant Research (IPK), Germany

## INTRODUCTION

The *Triticeae* tribe, which includes the important cereals bread wheat (*Triticum aestivum*), durum wheat (*T. turgidum*), barley (*Hordeum vulgare*) and rye (*Secale cereale*), provides a significant proportion of global nutrition. The grains of these important crops are produced on a structure called the inflorescence (Newton et al. 2011; Shiferaw et al. 2013), which contains a collection of grain‐producing florets that are arranged on a main stem (called a rachis), which develop within specialized branches, called spikelets that form on opposite sides of the rachis in an alternating phyllotaxy.

**Figure 1:**
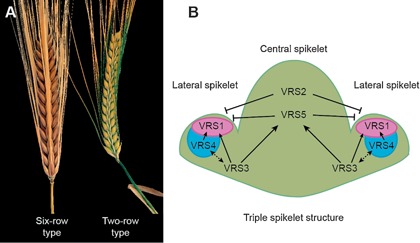
**Barley inflorescence architecture and known genetic pathways controlling row‐type phenotypes (A)** Mature inflorescences of two‐rowed and six‐rowed barley inflorescences. (**B)** Schematic diagram of a triple spikelet structure with a central spikelet and two lateral spikelets displaying known interactions of genetic pathways regulating row‐type architecture in barley. The purple and blue sections indicate localized expression of *Vrs1* and *Vrs4*, respectively, within the lateral spikelet primordia that encode proteins to suppress its fertility. Arrows indicate positive transcriptional effects. VRS1‐5 proteins all repress lateral spikelet development.

**Figure 2:**
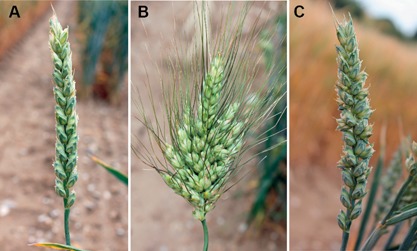
**Inflorescence architecture phenotypes of wheat (A–C)** Wheat inflorescences of **A**) a wild‐type plant (*cv*. Cadenza), **B**) a ‘Miracle wheat’ plant and **C**) a plant that forms paired spikelets. Plants were grown under field conditions, Norwich, United Kingdom.

Modifications in inflorescence architecture have been vital for cereal domestication by contributing to improved harvestability and yield, and use of genetic variation for key aspects of inflorescence development provides further opportunities to increase productivity (Doebley et al. 2006; Olsen and Wendel 2013). Among the *Triticeae*, research has focused largely on barley and wheat, which form very similar inflorescence architectures (Kirby and Appleyard 1987); however, there are important morphological differences between these two species. The lateral branches of barley form a triple spikelet structure composed of a central spikelet and two lateral spikelets, whose development is either suppressed to form a two‐rowed inflorescence type, or promoted to form a six‐rowed type (Figure 1A) (Brenchley 1920). Each spikelet is determinate and therefore contains only one floret. Conversely, in wheat, the inflorescence is composed of single spikelets that are indeterminate and produce multiple florets (Figure 2A) (Bonnett 1936). Furthermore, the wheat inflorescence is determinate and produces a terminal spikelet at the apex, whereas the barley inflorescence is indeterminate.

Our understanding of the genes and molecular processes that underpin inflorescence development has advanced significantly in recent years through improvements in sequencing capabilities that have made the barley and wheat genomes more accessible, and through the generation of mutant and mapping populations (Caldwell et al. 2004; Mascher et al. 2017; Krasileva et al. 2017; IWGSC 2018). This review summarizes the advancements in our understanding of the genes and molecular processes that regulate inflorescence architecture and development in wheat and barley, both through the investigation of key domestication‐related traits and through analysis of novel phenotypes and the use of new technologies.

## INVESTIGATION OF DOMESTICATION TRAITS TO UNCOVER GENES CONTROLLING INFLORESCENCE DEVELOPMENT IN BARLEY AND WHEAT

### Genetic regulation of inflorescence row‐type architecture in barley

A premier example of the advancements that have increased our understanding of the molecular regulation of inflorescence architecture in the *Triticeae* involves analysis of the two‐rowed inflorescence phenotype of barley, which has identified a multi‐faceted genetic pathway that suppresses development of the two lateral spikelets at a given node. Mutagenesis of two‐rowed barley identified at least five complementation groups of mutants that display either partial or complete fertility of lateral spikelets, which have been named *Vrs1‐5* (*six‐rowed spike1*) (Gustafsson and Lundqvist 1980; Lundqvist and Lundqvist 1988; Lundqvist et al. 1997). Loss‐of‐function alleles of these genes facilitate development and outgrowth of the lateral spikelets to form a six‐rowed inflorescence phenotype (Figure 1B).

A central gene within this network is *Vrs1*, a homeodomain‐leucine zipper I‐class homeobox transcription factor that suppresses development and growth of the lateral spikelets, which is thought to have arisen from a gene duplication event (Lundqvist et al. 1997; Komatsuda et al. 2007; Sakuma et al. 2010; Sakuma et al. 2013). *Vrs1* expression is restricted to the lateral spikelets at critical stages of inflorescence development when the lateral and central spikelets differentiate to form a triple mound structure, demonstrating that suppression of lateral spikelet growth is an early developmental decision (Komatsuda et al. 2007). This function of *Vrs1* is supported genetically by multiple forms of loss‐of‐function mutations in *Vrs1* being sufficient to confer a complete six‐rowed phenotype, as well as a semi‐dominant allele of *Vrs1* (*deficiens*) leading to extreme suppression of lateral spikelet fertility caused by a missense mutation that is predicted to prolong VRS1 protein function through later stages of inflorescence development (Komatsuda et al. 2007; Sakuma et al. 2017).

A central role for *Vrs1* in regulating row‐type phenotypes in barley is also highlighted by subsequent studies of other *vrs* mutants that display reduced expression of *Vrs1* during early stages of inflorescence development. For example, *Vrs1* expression is reduced in *vrs4* mutants that contain loss‐of‐function mutations within an orthologue of the maize *RAMOSA2* gene that encodes a LOB (LATERAL ORGAN BOUNDARIES) domain transcription factor (Koppolu et al. 2013). Expression of *Vrs4* is detected earlier than *Vrs1*, and is localized predominantly within the lateral spikelet primordia during early developmental stages, suggesting that VRS4 protein regulates lateral spikelet fertility by acting upstream of *Vrs1* (Koppolu et al. 2013). Similarly, *Vrs1* expression is reduced in *vrs3* mutants that contain mutations within a gene that encodes a histone demethylase, which is predicted to facilitate activation of *Vrs1* by removing repressive methyl marks and acting as a positive regulator of *Vrs4* (van Esse et al. 2017; Bull et al. 2017). Taken together, these findings indicate that *Vrs3* and *Vrs4* regulate row‐type architecture of the barley inflorescence by converging to positively regulate transcription of *Vrs1*.

Our understanding of the contribution that *Vrs1* plays during lateral spikelet development has been complemented by the identification of the gene that underpins the *vrs5* or *intermedium‐c* locus, which is a homologue of the maize domestication gene, *teosinte branched1* (*tb1*) (Lundqvist and Lundqvist 1988; Ramsay et al. 2011). Analysis of *Vrs1* and *Vrs5/Int‐c* alleles in cultivated barley revealed that particular combinations of alleles for these genes are maintained in two‐rowed and six‐rowed types, with the functional allele of *Vrs1* being partnered by the *int‐c.b* in two‐rowed cultivars, whereas non‐functional *Vrs1* alleles are partnered with the *Int‐c.a* allele (Komatsuda et al. 2007; Ramsay et al. 2011).

Although the function of these two *Vrs5/Int‐c* alleles remains unclear, comparative analysis of the predicted amino acid sequences for these two alleles (*Int‐c.a* and *int‐c.b*) with *TB1* homologues in wheat, maize, rice, and *Brachypodium distachyon* shows that the *Int‐c.a* allele is less evolutionarily conserved than the *int‐c.b* allele, suggesting that six‐rowed barley contains a deleterious *Vrs5/Int‐c* allele with reduced function (Doebley et al. 1995, 1997; Takeda et al. 2003; Ramsay et al. 2011; Dixon et al. 2018a). This conclusion is supported by the increased lateral spikelet fertility observed in lines that contain mutations preventing production of a functional VRS5/INT‐C protein (Ramsay et al. 2011). Less is known about the specific spatial and temporal expression of *Vrs5*, relative to *Vrs1*, *Vrs3* and *Vrs4*; however, *Vrs5* is expressed in the developing inflorescence and is reduced in *vrs3* mutants, which is consistent with a role in suppressing lateral spikelet development (Ramsay et al. 2011; van Esse et al. 2017; Bull et al. 2017). Interestingly, *Vrs1* expression is not significantly different in *vrs5*/*int‐c.5* loss of function mutants, relative to wild‐type, suggesting that *Vrs5* acts independently of *Vrs1* to suppress lateral spikelet fertility (Sakuma et al. 2013).

The final gene characterized to regulate inflorescence row‐type in barley is *Vrs2*, which encodes a SHORT INTERNODE transcriptional regulator that promotes a two‐rowed spikelet architecture by modulating hormone levels during inflorescence development (Youssef et al. 2017). In *vrs2* loss‐of‐function mutants, enlarged and fertile lateral spikelets form at the central region of the inflorescence, and additional supernumerary spikelets form at the basal region (Gustafsson and Lundqvist 1980; Lundqvist and Lundqvist 1988; Youssef et al. 2017). Expression of *Vrs2* is not confined to lateral spikelet primordia, as for *Vrs1* and *Vrs4*, but it is expressed more generally throughout spikelets at the triple mound stage, within floret primordia and in tiller buds (Youssef et al. 2017). Interestingly, *Vrs2* expression was significantly higher in the basal and central regions, relative to the apical regions of the inflorescence, which is consistent with the location of the spikelet architecture phenotypes observed in *vrs2* mutants (Youssef et al. 2017).

This gradient of *Vrs2* expression influences the expression of genes that regulate biosynthesis and metabolism of auxin and cytokinin, such that the opposing apical to basal gradients of these hormones in wild‐type inflorescences are disrupted in *vrs2* mutants (Youssef et al. 2017). Absence of *Vrs2* also perturbs expression of gibberellin (GA) biosynthesis genes, which is thought to contribute to the delay in inflorescence development observed in *vrs2* mutants (Youssef et al. 2017). This conclusion is supported by a previous study demonstrating that reduced levels of GA extend the duration of inflorescence development and delay flowering in barley (Boden et al. 2014). These studies implicate hormones as important contributors to spikelet row‐type architecture and inflorescence development in barley, which is supported by genome‐wide expression analysis in *vrs3* mutants that showed differential expression of genes involved in cytokinin and jasmonic acid metabolism, relative to wild‐type plants (Bull et al. 2017; van Esse et al. 2017; Youssef et al. 2017). Taken together, these results suggest that adjustments to the levels and distribution of hormones, during inflorescence development, can be used to generate diverse inflorescence architectures in barley (Boden 2017).

Identification of genes that underpin each of the loci known to regulate row‐type architecture in barley provides an opportunity to investigate the agronomic significance of the triple spikelet structure (Figure 1B). Infertile lateral spikelets are common to all wild barley and provide an evolutionary advantage by facilitating penetrance of the shattered grain into soil, and assisting grain dispersal, *via* zoochory (von Bothmer et al. 1995; reviewed in Pourkheirandish and Komatsuda 2007). However, recent evidence suggests that formation of the lateral spikelets may also influence pre‐harvest as well as grain dispersal and germination traits, such as grain development. For example, the extremely suppressed lateral spikelet development of the *deficiens* mutant facilitates enlarged grain size for the central spikelet, and introgression of mutant *vrs3* alleles into six‐rowed backgrounds that contain loss‐of‐function *vrs1* and *vrs5* alleles improves uniformity of grain size produced by the lateral spikelets, relative to the central spikelet (Bull et al. 2017; Sakuma et al. 2017). It remains to be determined how these alleles of the row‐type architecture genes affect grain size; however, this effect may be associated with altered assimilate partitioning or reduced competition between the central and lateral spikelets (Sakuma et al. 2017). Nonetheless, these results suggest that there is potential to use variant alleles of the row‐type genes, or combinations of alleles for multiple genes to improve grain yield or grain processing‐related traits.

### Genetic regulation of inflorescence compactness in wheat and barley

In wheat, a prime example of the advances made in our understanding of the genetic regulation of inflorescence development comes from investigation of the *Q* gene, which encodes an *AP2‐like* transcription factor that regulates important aspects of inflorescence development. Transformation of the *Q* gene on chromosome 5A from the ‘*q*’ allele of ancestral wheat to the ‘*Q*’ allele of modern wheat has conferred many important domestication traits, including a non‐brittle rachis, free‐threshing grains, a compact spike, and altered glume shape (Faris et al. 2003; Simons et al. 2006).

The free‐threshing characteristic provided by *Q* has been particularly significant because it facilitates removal of the hull (glumes, palea and lemma) from the grain, relative to the non‐free‐threshing ancestral wheat lines, including emmer (*T. dicoccoides* Körn.) and einkorn (*T. boeoticum* Boiss.) that carry the *q* allele. The *Q* allele also prevents grain loss that occurs in emmer and einkorn due to brittle inflorescences that shatter into spikelets at maturity (Salamini et al. 2002). It has also been shown recently that the *Q* allele increases grain yield, grains m^−2^ and thousand grain weight, while exhibiting a decrease in grain per spike, relative to plants with the *q* allele (Xie et al. 2015; Xie et al. 2018).

Analysis of *q* and *Q* alleles and the structure of the encoded proteins has provided insights into the regulation of *Q* and its contribution to the inflorescence traits, which is thought to involve two possible mechanisms of regulation: firstly, a missense mutation that alters the amino acid sequence, and secondly a single nucleotide change that affects a microRNA (miRNA) binding site. The effects of the *Q* gene are dosage dependent, with increased transcript levels being associated with corresponding levels of inflorescence compactness and reduced plant height (Muramatsu 1963; Simons et al. 2006). Consistent with the dosage effect, it has been shown that the expression of *Q* is higher than *q*; however, the alleles share similar developmental expression patterns, with expression being highest at the early stages of inflorescence development and lower at later stages, indicating that *Q* plays a key role in the initial stages of inflorescence development (Simons et al. 2006; Debernardi et al. 2017).

A possible cause for the reduced expression has been linked to the non‐synonymous change of valine to isoleucine, where *q* genotypes contain a valine and the *Q* genotypes contain an isoleucine (Simons et al. 2006). The substitution of isoleucine for valine diminishes the ability of the full‐length Q protein to form a homo‐dimer, suggesting that the differences in *Q* gene expression in ancestral wheat relative to modern cultivars could be due to the lack of homodimer formation (Simons et al. 2006). However, subsequent analysis of the *Q* and *q* protein structure indicates that the V329I mutation does not affect the protein 3D structure, and so it is unlikely to affect protein activity (Sormacheva et al. 2015).

**Figure 3:**
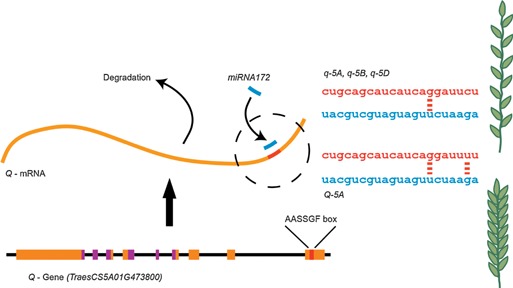
**Molecular regulation of *Q* gene activity in wheat** Diagram showing post‐transcriptional regulation of the *Q* gene by binding of miR172 (blue) to target the mRNA for degradation, with the *Q* allele being less susceptible to miR172 degradation due to a G:U wobble, resulting in a more compact spike. Genomic regions encoding the AP2 domains are shown in purple and the AASSGF box which is the miRNA binding site is shown in red. Figure adapted from Debernardi et al. (2017).

More recently, the mechanism for *Q* regulation was shown to include a role for the microRNA miR172, which is a small non‐coding RNA that post‐transcriptionally regulates AP2‐like transcription factors to effect important developmental processes (Chen 2004; Debernardi et al. 2017; Greenwood et al. 2017). Both the *Q* and *q* alleles contain an AASSGF box, which is the binding site for miR172, and mutation of miR172 and its target site in exon 10 of *Q* leads to mis‐regulated expression of the *Q* gene (Figure 3) (Aukerman and Sakai 2003; Chen 2004; Chuck et al. 2007). The importance of the miRNA172 binding site in regulating *Q* expression was supported recently by identification of a novel mutation in the miR172 binding region identified in plants that resembled mutants with an increased copy number and expression of *Q*, highlighting that mutation of the miRNA binding region alone is sufficient to affect gene function (Greenwood et al. 2017). Similarly, increased expression of the miR172 (tae‐miR172) precursor was shown to correlate with decreased *Q* gene expression, demonstrating that the *Q* mutation is not sufficient to completely negate its downregulation by miR172 (Liu et al. 2018). Moreover, a dual‐luciferase sensor system was used to demonstrate that the *Q* allele has a weaker miR172 target site than *q*, which reduced the mRNA cleavage efficiency (Debernardi et al. 2017).

The *Q* allele carries a G:U wobble in the 5 prime end of the miRNA binding site instead of the strong G‐C pairing observed in the *q* allele to miR172 (Figure 3). This mutation reduces the energy of the interaction and decreases the ability of miRNA to repress gene activity (Liu et al. 2014; Debernardi et al. 2017). In wheat varieties where the A genome carries the *Q* allele and is compared to the *q‐5B* and *q‐5D*, *Q‐5A* shows a 36‐fold decrease in miR172‐mediated degradation of product, despite having similar transcript levels (Debernardi et al. 2017). The recent advances in understanding regulation of the *Q* gene show that the transition from *q* to *Q*, during wheat domestication, was a gain‐of‐function event, with the mutation in the miRNA binding site preventing the degradation of *Q* mRNA to subsequently increase Q protein levels.

The function of the Q protein has been investigated, with promising results pointing to a role as a transcriptional repressor that interacts with co‐repression factors (Liu et al. 2018). Analysis of co‐expression profiles of the *Q* gene and *TOPLESS* (*TaTPL*), in combination with yeast two hybrid screen and a firefly luciferase complementation imaging assay, showed that Q protein is likely to function as a transcriptional co‐repressor in partnership with *Ta*TPL (Liu et al. 2018). This finding is supported by the presence of two EAR motifs in *Q* that are known to interact with TPL/TRP transcriptional co‐repressor (Krogan et al. 2012; Liu et al. 2018).

Interestingly, analysis of the *Q* gene in wheat has been complemented recently by studies in barley that have shown synonymous and non‐synonymous mutations in the barley *Q* paralog, *HvAP2*, affect compactness of the inflorescence and plant height (Houston et al. 2013; Skov Kristensen et al. 2016). Investigation of the *ZEOCRITON (Zeo)* gene has demonstrated that its activity has a major impact on inflorescence density, with *Zeo1.b* plants having inflorescences twice as dense as wild‐type plants. The responsible genomic region was narrowed to a gene encoding a transcription factor with two *AP2* DNA‐binding domains, in addition to a miR172‐binding site, and was therefore named as *HvAP2*. The mutation in *HvAP2* that increased density of the inflorescence was concluded to be in the miR172 binding site, which reduced the cleavage efficiency of *HvAP2*. Interestingly, the authors showed that miR172 appears to function predominantly during the early stages of inflorescence development, but not in the later developmental stages, indicating a critical role during the transition from spikelet formation to awn initiation and internode elongation (Houston et al. 2013).

## BEYOND DOMESTICATION: A NEW BRANCH OF UNDERSTANDING FOR INFLORESCENCE ARCHITECTURE REGULATION

Analysis of traits that have contributed to wheat and barley domestication were recently complemented by investigation of modified architectures to uncover new genes that regulate inflorescence development and provide potential strategies for improved grain productivity (Dobrovolskaya et al. 2014; Poursarebani et al. 2015; Boden et al. 2015). For example, investigation of the highly branched inflorescences of tetraploid ‘Miracle wheat’ and ‘Compositum‐barley’ plants led to the identification of *APETALA2*/*ETHYLENE RESPONSIVE FACTOR* (*AP2*/*ERF*) transcription factor, which is homologous to the *FRIZZY PANICLE* (*FZP*) and *BRANCED SILKLESS* (*Bd1*) genes of rice and maize, respectively (Chuck et al. 2002; Komatsu et al. 2003; Poursarebani et al. 2015). Recessive missense mutations in the *BRANCHED HEAD1* (*TtBH1*) and *COMPOSITUM 2* (*COM2*) genes of tetraploid wheat and barley mutants, respectively, facilitate development of inflorescence‐like structures at rachis nodes where a spikelet typically forms, suggesting that BH1 and COM2 function is required for the axillary meristems to obtain a spikelet identity (Poursarebani et al. 2015). This conclusion is further supported by analysis of hexaploid wheat inflorescences that form multi‐row supernumerary spikelets characterized by the development of multiple spikelets at a given rachis node, which contain frameshift mutations or deletions within *BH1*. However, in this case, the gene was named *WFZP* (for wheat *FZP*) (Sharman 1967; Dobrovolskaya et al. 2014). Mutations in *WFZP* are also suspected to cause the four‐rowed spikelet phenotype of tetraploid and hexaploid wheats that are characterized by the formation of two adjacent spikelets at a given node, as the locus responsible for this phenotype has been mapped to a syntenic region on chromosome 2A (Zhang et al. 2013). These studies demonstrate, therefore, that *BH1*/*FZP*/*COM2* is a critical gene required to promote formation of the unbranched spike inflorescence structure of the *Triticeae*, and that loss‐of‐function mutations in key genes that promote spikelet meristem identity could be used to develop wheat and barley plants with elaborate branching phenotypes.

Modulation of spikelet meristem identity gene activity to form more elaborate inflorescence branching in wheat has also been demonstrated recently by investigation of paired spikelets. These are supernumerary spikelets, characterized by the formation of two spikelets at a given node with a secondary spikelet forming immediately adjacent to and below the typical primary spikelet (Sharman 1944, 1967; Boden et al. 2015; Dixon et al. 2018a). Analysis of this trait, in an advanced mapping population, identified 18 contributing quantitative trait loci (QTL), suggesting that there are multiple genes that contribute to spikelet development (Boden et al. 2015).

**Figure 4:**
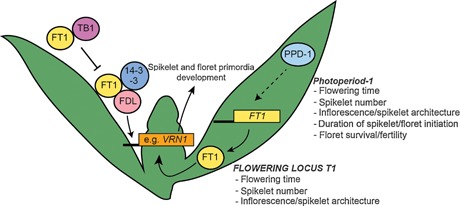
**Regulation of inflorescence architecture and development in wheat by components of the floral promoting pathway** Diagram illustrating the roles of known components of the floral promoting pathway in determining key inflorescence architecture and development traits in wheat. Photoperiod‐1 (PPD‐1) helps promote expression of *FLOWERING LOCUS T1* (*FT1*), which generates FT protein in the leaf that is transferred to the shoot apical meristem where it interacts with FDL (FD‐Like) and 14‐3‐3 proteins to form a floral activating complex (FAC), which induces expression of meristem identity genes, such as *VERNALIZATION1* (*VRN1*). TEOSINTE BRANCHED1 (TB1) interacts with FT1 and suppresses its ability to promote expression of meristem identity genes, possibly by suppressing FT1 from forming part of the FAC. Proteins (PPD‐1, FT1, TB1, FDL, 14‐3‐3) are indicated in solid circles, and genes (*FT1* and *VRN1*) are shown using solid rectangles. Text alongside *Ppd‐1* and *FT1* notes known traits affected by these genes.

The most significant of these QTLs was shown to be underpinned by *Photoperiod‐1* (*Ppd‐1*), which is a pseudo‐response regulator that regulates photoperiod responsive flowering pathways in wheat and barley (Turner et al. 2005; Beales et al. 2007; Shaw et al. 2013; Boden et al. 2015). It was demonstrated that *Ppd‐1* influences paired spikelet development by modulating the expression of *FLOWERING LOCUS T1* (*FT1*), which is a central integrator of flowering that promotes flowering under inductive photoperiods by activating expression of meristem identity genes within the shoot apical meristem (Corbesier et al. 2007; Tamaki et al. 2007; Boden et al. 2015) (Figure 4). Paired spikelets formed under genetic and environmental conditions that elicit a weak flowering signal, including loss‐of‐function mutations in *Ppd‐1* and *FT1*, or growth under short‐day photoperiods, whereas conditions that promote a strong flowering signal suppressed paired spikelet development (Boden et al. 2015).

Gain‐of‐function photoperiod‐insensitive alleles of *Ppd‐1* were shown recently to reduce the number of spikelets that form on an inflorescence, which is caused by a shortened duration of early developmental stages that can be only partially compensated by an increased rate of spikelet initiation (Ochagavía et al. 2018) (Figure 4). Moreover, *FT‐B1* (*FT1* from the B genome of bread wheat) acts downstream of *Ppd‐1* to influence spikelet number, with loss of *FT‐B1* resulting in increased spikelet number, in a thermally‐responsive manner (Dixon et al. 2018b; Finnegan et al. 2018) (Figure 4). FT1‐dependent control of spikelet architecture was also supported by recent analysis of *TEOSINTE BRANCHED1* (*TB1*; orthologue of *Vrs5*) function in wheat, which facilitates paired spikelet development, in a dosage dependent manner, by interacting with *FT1* and reducing FT1‐dependent activation of spikelet meristem identity genes (Dixon et al. 2018a) (Figure 4). Investigation of plants that were tetrasomic for chromosome 4D and transgenic lines that expressed *TB1* at higher levels showed that increased dosage of *TB1* promotes paired spikelet development, and allelic variation for *TB1* was shown to be associated significantly with paired spikelets in modern wheat cultivars (Dixon et al. 2018a). Taken together, these results show that transcriptional and post‐translational regulation of flowering signals can alter inflorescence architecture, which has potential to be used as a mechanism for increasing spikelet and floret numbers in wheat.

A unique aspect of inflorescence architecture, in wheat, is the formation of indeterminate spikelets that produce multiple florets, and improvement of floret fertility is a promising strategy for increasing grain production (Kirby 1988; Reynolds et al. 2009). Floret primordia develop within spikelets during early stages of inflorescence development to peak at 7–12 primordia at the green anther stage; however, a large proportion of these primordia abort so that only 3–5 florets survive to produce grain (Kirby 1988; Guo and Schnurbusch 2015; González‐Navarro et al. 2015).

Analysis of the dynamics of floret development and abortion have demonstrated that genetic variation for floret fertility exists in modern wheat, and that floret survival could be improved by two methods: (i) extending the period between completion of floret primordia development and initiation of floret degeneration, and (ii) optimizing assimilate distribution to spikelets and florets, which is supported by floret fertility being associated with ovary size at anthesis (Guo and Schnurbusch 2015; González‐Navarro et al. 2015; Guo et al. 2016; Guo et al. 2017; Prieto et al. 2018).

Although the genes that regulate floret survival remain largely unknown, photoperiod insensitive *Ppd‐1* alleles, involved in promoting early flowering, reduce the number of fertile florets by effecting the developmental phase during which florets form, the rate of floret appearance and floret survival (González et al. 2005; Prieto et al. 2018) (Figure 4). Moreover, a complex QTL analysis of multiple floret fertility‐related traits identified 52 loci, including regions on chromosomes 5B, 5D and 6A, which associate with traits including maximum floret primordia, grain number per spikelet, floret loss and grain survival (Guo et al. 2017). Interestingly, one QTL associated with floret primordia loss was identified on chromosome 2A, in proximity to the wheat homologue of *Vrs1*, suggesting that *Vrs1* may have a conserved role in barley and wheat for suppressing floret development (Guo et al. 2017). These studies highlight the potential to improve grain production in wheat by harnessing genetic variation of floret fertility‐related traits, and indicate that dissection of the genetic pathways from one of the *Triticeae* may benefit optimization of inflorescence development for other members of the tribe.

To complement the genetic advancement in our understanding of the genetic regulation of inflorescence architecture, recent improvements in the sequence of the barley and wheat genomes have facilitated the use of genome‐wide transcriptome analysis to identify novel regulators of inflorescence development (Digel et al. 2015; Pearce et al. 2015; Borrill et al. 2016; Mascher et al. 2017; Wang et al. 2017; IWGSC 2018; Ramirez‐Gonzalez et al. 2018). For example, in barley, analysis of multiple stages of inflorescence development identified genes important for the vegetative‐to‐reproductive transition, including the MADS box transcription factors *BARLEY MADS BOX1* (*BM1*) and *VEGETATIVE TO REPRODUCTIVE TRNASITION2* (*VRT2*) that are repressed during the transition, and genes including *SQUAMOSA PROMOTER BINDING‐LIKE PROTEIN4* (*SPL4*), *KNOTTED1* (*KN1*) and *SUPPRESSOR OF CONSTANS1* (*SOC1*) that were activated (Digel et al. 2015). In addition to floral development genes, genes involved in carbohydrate transport, nitrate transport and hormone signaling (e.g. *SWEET15*, *NITRATE TRANSPORTER1* and *GA2oxidase*) were also upregulated during the floral transition; interestingly, these genes were among those mis‐expressed in *vrs3* and *vrs2* mutants (Digel et al. 2015; Bull et al. 2017; Youssef et al. 2017). In addition, genes upregulated in the shoot apical meristem (SAM) in a photoperiod‐ and/or *Ppd‐1‐­*dependent manner were also identified, including a member of the *FLOWERING LOCUS T‐like* family, *FT2*, floral homeotic genes, including *SEPALLATA1* (*SEP1*), *SEP3*, *PISTILLATA* (*PI*) and *APETALA3* (*AP3*), as well as MADS box transcription factors, including *VERNALIZATION1* (*VRN1*), *BM3* and *BM8* (Digel et al. 2015). Interestingly, homologues of *VRN1*, *BM3* and *BM8* were also upregulated in wheat SAMs, in a *Ppd‐1*‐dependent manner (named *VRN1*, *AGL10* and *AGL29*, respectively), and were upregulated in SAMs of barley lines that contain null alleles for *EARLY FLOWERING3* (Boden et al. 2014; Boden et al. 2015).

Similar transcriptome profiling was also performed in wheat to identify genes whose expression is associated with key yield‐related traits, including spikelet and floret numbers (Wang et al. 2017). While this analysis used a less conventional nomenclature of developmental stages, it did identify *TaPAP2* (a wheat orthologue of the rice gene, *PAP2*, which is more commonly referred to as *AGLG1* [*AGAMOUS‐LIKE GENE1*] in wheat), *WFZP*, *LAX PANICLE1* (*LAX1*) and *TERMINAL FLOWER1* (*TFL1*) as genes whose expression was associated significantly with spikelet number (Yan et al. 2003; Zhao et al. 2006; Boden et al. 2015; Wang et al. 2017). Over‐expressing *PAP2/AGLG1* decreased inflorescence length, spikelet and floret numbers in a way consistent with its role as a floral activator, whereas overexpression of *TFL1* facilitated development of additional spikelets and florets that supports a function for TFL1 in maintaining meristem dormancy and delaying the vegetative‐to‐reproductive transition (Wang et al. 2017). Interestingly, Wang and colleagues also overexpressed a homologue of *HvHOX2*, which reduced spikelet and floret numbers and inflorescence length to indicate a possible divergence in function relative to barley (Sakuma et al. 2010; Sakuma et al. 2013; Wang et al. 2017).

Taken together, these studies highlight the potential for advanced sequencing capabilities to accelerate identification of genes that have important roles during inflorescence development in wheat and barley, and to identify molecular targets for increased grain production by discovering genes whose expression is associated with key yield‐related traits.

## FUTURE PERSPECTIVES

Research during the last decade has advanced considerably our understanding of the genetic regulation of inflorescence development in wheat and barley, and insights from these studies point towards future research providing equally fruitful progress. For example, analyses of multiple *Vrs* genes have identified extensive natural variation for alleles within cultivated barley that include numerous missense mutations, which provide a rich resource to perform functional characterization of the encoded proteins and domains contained therein (Komatsuda et al. 2007; Ramsay et al. 2011; Koppolu et al. 2013; Youssef et al. 2017).

The opportunity to benefit from an improved understanding of protein function has been demonstrated through analysis of *Slender1* (*Sln1*), which encodes the barley DELLA protein that controls GA‐dependent growth responses (Chandler et al. 2002). Analysis of lines that contain intragenic mutations within the dwarf allele of *Sln1* revealed amino acids that specifically control size of the shoot apical meristem and the number of florets that form on a barley inflorescence. This illustrates the possibility to improve yield‐related traits by an advanced understanding of gene function (Chandler and Harding 2013; Serrano‐Mislata et al. 2017). Similarly, the regulation of the *Q* gene in wheat and VRS1 protein in the *deficiens* mutant indicates that post‐transcriptional and post‐translational regulation of gene and protein activity plays an important role during inflorescence development, which could be an important area of future research (Debernardi et al. 2017; Greenwood et al. 2017; Sakuma et al. 2017).

Identification of the *Vrs1‐5* genes in barley also presents an opportunity to investigate the broader role of row‐type genes during inflorescence and grain development by combining alleles to make double or triple mutant lines (Zwirek et al. 2018). For example, introgression of the mutant *vrs3* allele into the cultivar Morex, which contains the six‐rowed *Vrs1* and *Vrs5* alleles, improved uniformity of grain size by increasing the width and area of grains produced by the lateral spikelets (Bull et al. 2017). Analysis of spikelet, floret and grain development in other double and triple mutant combinations may reveal further information about the function of the *Vrs* genes and their contribution to key agronomic traits. Similarly, comparative analysis of the transcriptome data from the *vrs* mutants may help identify downstream genes that are commonly affected by the six‐rowed alleles, which may help uncover more about the molecular regulation of spikelet architecture (Bull et al. 2017; van Esse et al. 2017; Youssef et al. 2017).

The genetic relatedness of wheat and barley, in combination with the altered inflorescence and spikelet meristem determinacy of these two species, provides a unique opportunity to determine the genes and biological processes that contribute to the diverse inflorescence architecture of cereals. For example, in barley, reduced function of *Vrs5*/*TB1* facilitates development of the lateral spikelets, whereas in wheat, increased dosage of *TB1* promotes formation of secondary spikelets, which could be interpreted to mean that *Vrs5*/*TB1* suppresses axillary spikelet development in barley but promotes more elaborate branching in wheat (Ramsay et al. 2011; Dixon et al. 2018a). An alternate explanation is that *Vrs5*/*TB1* promotes meristem dormancy, which influences the transition of meristem determinacy and outgrowth of lateral organs. In this scenario, an increased dosage of *TB1* in wheat suppresses the transition of a lateral branch meristem into a spikelet meristem to facilitate development of a short branch composed of two spikelets, whereas in barley, reduced function of *Vrs5*/*TB1* disrupts the dormancy of the lateral spikelet primordia to facilitate development and growth of fertile lateral spikelets.

Similar comparisons of reciprocal gene function in wheat and barley, such as *Vrs1* and *HOX2*, will advance our understanding of the genetic regulation of inflorescence development within each species, while also contributing to our knowledge about the biological processes that contribute to the diverse architectures of wheat and barley (Komatsuda et al. 2007; Sakuma et al. 2013; Wang et al. 2017; Sakuma et al. 2018). This investigation will be facilitated by the mutant populations generated recently for barley, tetraploid wheat and hexaploid wheat, which can be used in a forward genetics approach to identify novel inflorescence development genes, and in a reverse genetics approach to investigate the function of known inflorescence architecture genes in the respective species (Caldwell et al. 2004; Krasileva et al. 2017). The potential for a reverse genetics approach to provide vital information is supported by the discovery of *Vrs4* as a homologue of *RAMOSA2*, which has a well‐characterized role in regulating inflorescence architecture in maize (Bortiri et al. 2006; Gallavotti et al. 2010; Koppolu et al. 2013).

## CONCLUSIONS

In conclusion, analysis of the genes that underpin key domestication‐related traits of inflorescence architecture in barley and wheat has contributed significantly to our understanding of the molecular processes that regulate inflorescence development in the *Triticeae*. This research is now being extended to investigate the genetic basis of modified inflorescence architectures, and further research that uses new genomic and mutant resources promises to provide valuable insights into the genes that control spikelet and floret development. Continued investigation of the genetic regulation of inflorescence architecture in the *Triticeae* promises to be a rich area of research to pursue fundamental knowledge about plant reproductive development, while also providing valuable insights that can be used by breeders to optimize key yield‐related traits for increased grain production in wheat and barley.
